# Gaps in awareness of metabolic liver disease among high-risk populations in the United States

**DOI:** 10.1097/HC9.0000000000001065

**Published:** 2026-10-19

**Authors:** Trenton M. White, Lila Neal, Scott Isaacs, Sonal Kumar, Melina I. Manolas, Silvana Pannian, Fasiha Kanwal, Leire Agirre-Garrido, Jeffrey V. Lazarus

**Affiliations:** 1 CUNY Graduate School of Public Health and Health Policy (CUNY SPH), New York, New York, USA; 2 Barcelona Institute for Global Health (ISGlobal), Hospital Clínic, University of Barcelona, Barcelona, Spain; 3 Department of Medicine, Emory University School of Medicine, Atlanta, Georgia, USA; 4 Department of Medicine, Division of Gastroenterology and Hepatology, New York-Presbyterian Hospital/Weill Cornell Medical Center, New York, New York, USA; 5 Department of Medicine, Division of Endocrinology, NewYork-Presbyterian Hospital/Weill Cornell Medical Center, New York, New York, USA; 6 Section of Adult and Pediatric Endocrinology, Diabetes and Metabolism, University of Chicago, Chicago, Illinois, USA; 7 Department of Medicine, Section of Gastroenterology and Hepatology, Baylor College of Medicine, Houston, Texas, USA; 8 VA Health Services Research and Development Service, Center for Innovations in Quality, Effectiveness, and Safety, Houston, Texas, USA; 9 Michael E DeBakey VA Medical Center, Houston, Texas, USA

**Keywords:** cirrhosis, health communication, health literacy, MASLD, steatotic liver disease

## Abstract

**Figure:**
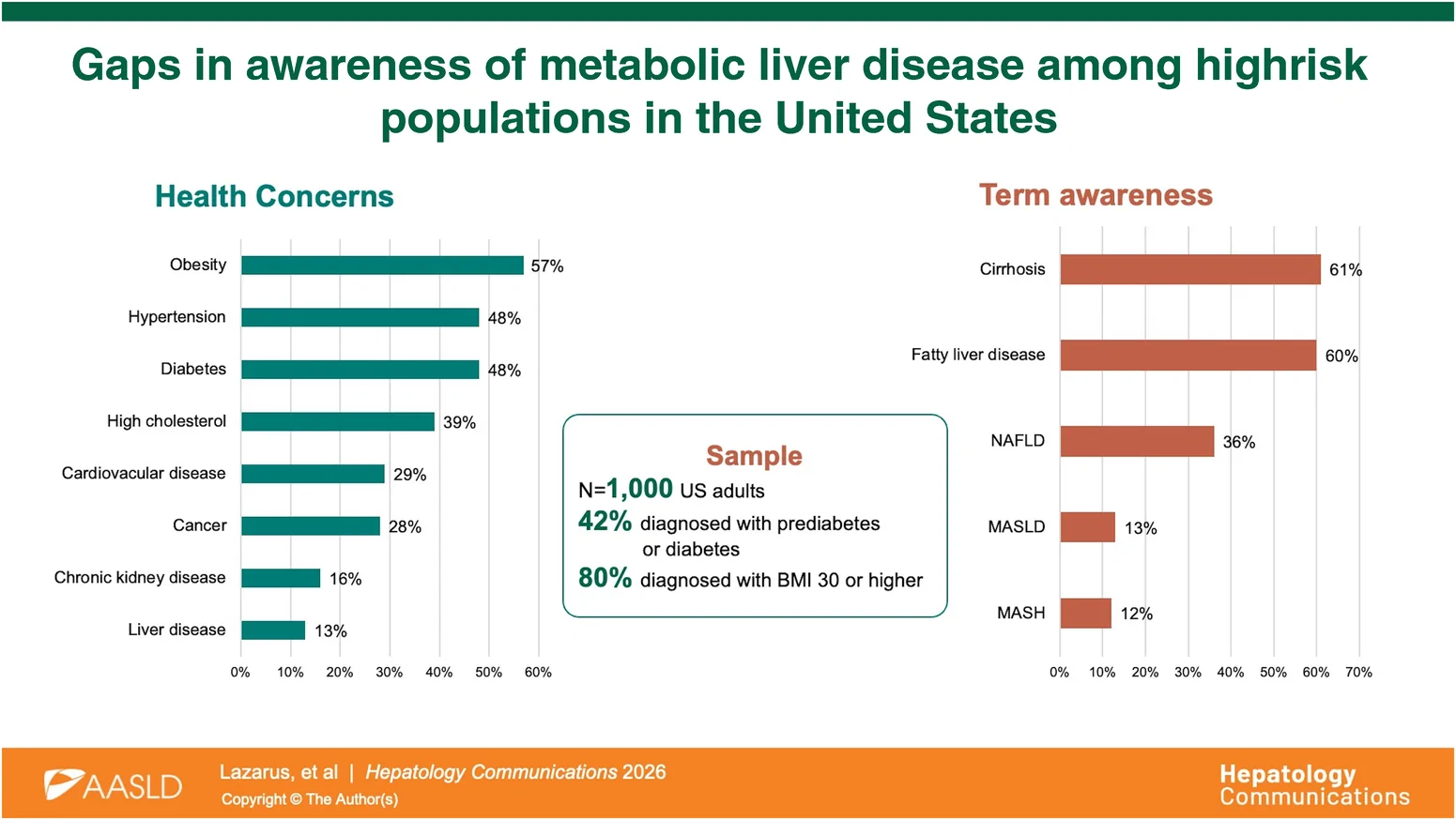

## INTRODUCTION

With an estimated global prevalence of over 30%, metabolic dysfunction–associated steatotic liver disease (MASLD) presents a threat to public health and was recognized as such in the 2026 World Health Assembly resolution on steatotic liver disease.^1^,^2^ MASLD and its more progressive form, metabolic dysfunction–associated steatohepatitis (MASH), are characterized by the accumulation of excessive fat in the liver, predisposing to liver fibrosis and cirrhosis.^3^ Despite this high morbidity and association with type 2 diabetes (T2D), overweight and obesity, as well as cardiovascular disease and hepatic and extra-hepatic forms of cancer,^3^ awareness of MASLD remains very low among the general population.^4^,^5^,^6^

People living with higher-risk “indicator” conditions, such as obesity and T2D,^7^ do not often perceive themselves as being at-risk for MASLD.^3^ Despite recent advances in therapeutics for MASLD and MASH, including the emergence of approved pharmacologic therapies for patients with noncirrhotic MASH and moderate-to-advanced fibrosis (F2–F3), awareness of MASLD remains low among primary care providers,^4^,^8^ who frequently serve as the first line of detection.^6^

While previous studies have highlighted the low awareness of MASLD among the general population,^4^ the data available on MASLD awareness among specific at-risk groups remain limited. Research on this awareness gap can generate evidence for health sector investments, including in education and targeted communication.^3^,^6^ Therefore, the aim of this study was to assess awareness and the level of health concern of liver-related and key cardiometabolic conditions for which this population is at elevated risk.

## METHODS

### Study design and sample recruitment

This study employed a cross-sectional survey design using an online panel administered by YouGov. A total of 3010 adult residents of the United States (US) were initially recruited, who were then matched down to the representative sample frame described below, resulting in 2581 US adults. From this matched dataset, a subsample of 1000 respondents meeting the eligibility criteria (ie, body mass index ≥27), prediabetes, or T2D was selected for analysis (Supplemental Table S1, https://links.lww.com/HC9/C511).

### Sampling frame construction

Respondents were matched to a sampling frame constructed from the 2023 American Community Survey (ACS) 1-year sample. The frame was developed using stratified sampling based on key demographic characteristics, including age, race, and education. Within each stratum, selection was conducted using weighted sampling with replacement, applying person-level weights from the ACS public use file. Matching variables included gender, age, race, and education to ensure alignment between the survey sample and the broader US population. Surveys were fielded from February 5, 2026, through February 12, 2026.

### Questionnaire

The survey questionnaire of 5 items asked respondents about their familiarity with liver disease terms (liver cirrhosis, fatty liver disease, non-alcoholic fatty liver disease (NAFLD), liver fibrosis, MASLD, and MASH). It evaluated personal health concerns (for obesity, diabetes, hypertension, and liver disease), previous MASLD diagnosis, and whether they are currently seeing a health care provider and whether certain strategies for the management of metabolic conditions had been mentioned by their doctors.

### Analysis

Descriptive statistics of the data are presented in Table 1 and Supplemental Table S2, https://links.lww.com/HC9/C511.

**TABLE 1:** Familiarity with liver disease terms by subsamples

Terms	Full sample (n=1000) (%)	BMI 30+ (n=804) (%)	Living with type 2 diabetes or prediabetes (n=441) (%)	Reported MASLD diagnosis* (n=68) (%)	Knowing parent with MASLD* (n=18) (%)	Knowing partner with MASLD* (n=22)(%)	Knowing friend with MASLD* (n=62) (%)	Knowing colleague with MASLD* (n=9) (%)
Liver cirrhosis	61	59	67	85	79	70	77	77
Fatty liver disease	60	59	64	94	91	80	92	63
NAFLD	36	36	38	80	63	62	70	47
Liver fibrosis	24	23	26	51	48	25	52	53
Metabolic dysfunction–associated steatotic liver disease (MASLD)	13	13	15	27	40	14	64	35
Metabolic dysfunction–associated steatohepatitis (MASH)	12	11	15	26	29	20	34	44

*Note:* Items with an asterisk (*) are from the question “Please indicate whether the following statement is true for you: I know someone who has been diagnosed with MASLD (also called fatty liver disease or NAFLD.”

### Ethics

The YouGov research protocol was reviewed and approved for Federalwide Assurance (FWA) by the Department of Health and Human Services (DHHS) under FWA number FWA00010960. All participants provided study-specific informed consent before completing the survey.

## RESULTS

In the full sample, awareness of cirrhosis (61% of n=1000) was highest, followed by fatty liver disease (60%), and dropped for more specific terms such as NAFLD (36%), liver fibrosis (24%), MASLD (13%), and MASH (12%) (Table 1). Awareness was slightly higher across these terms among respondents with T2D or prediabetes compared with those living with overweight/obesity (body mass index ≥ 27), and markedly higher among those reporting a previous diagnosis of MASLD. Individuals with indirect exposure to MASLD (eg, knowing a parent, partner, or friend) also showed elevated awareness compared to the general population, though recognition of MASLD and MASH remained inconsistent across groups.

The most reported health concerns across the full sample were obesity (57%), hypertension (48%), and diabetes (48%), while concern about liver disease was 13% (Supplemental Table S2, https://links.lww.com/HC9/C511). Respondents with obesity and those with T2D or prediabetes reported higher concern for cardiometabolic conditions, particularly diabetes (81% among those with diabetes/prediabetes). Those living with MASLD reported elevated concern across multiple conditions, including obesity (69%) and liver disease (39%). A similar pattern was observed among those with indirect exposure to MASLD, with relatively low prioritization of liver disease despite higher overall health concern profiles.

## DISCUSSION

This study identified an incongruity between awareness and risk profile among individuals at risk for liver-related and cardiometabolic conditions. Higher awareness of fatty liver disease than MASLD and MASH may reflect delayed uptake following the 2023 nomenclature change from NAFLD.^9^ The high recognition of cirrhosis may reflect its public association with both alcohol-associated harm and advanced disease severity.^6^

MASLD is reversible, particularly in its early phases, underscoring the importance of timely detection and intervention to prevent progression to fibrosis, cirrhosis, and associated complications.^3^ The observed gaps in MASLD/MASH awareness highlight the need for targeted education interventions among at-risk groups, including individuals with obesity and T2D, that extend beyond general liver disease messaging to explicitly address metabolic risk pathways, disease reversibility, and the importance of early screening and lifestyle modification. While general concern for cancer remained stable near 28% across most groups, immediate metabolic conditions like obesity and diabetes dominated their concerns. This pattern underscores a critical public health challenge in individuals navigating complex, daily metabolic management: long-term or silent risks such as cancer or progressive liver disease may be deprioritized in favor of top-of-mind metabolic diagnoses. Implementation of the World Health Organization Best Buys, which, while not explicitly mentioning MASLD, do address many of its risk factors, from alcohol to poor diet. They are important country-level policies that can contribute to changing the course of MASLD and reducing its prevalence.^10^

This study is subject to limitations, including reliance on self-reported data (eg, body mass index, diabetes, and MASLD status), potential selection bias inherent to online panel surveys despite weighting adjustments, such as exposure to health-related surveys or online ads for MASLD/MASH treatment, and the absence of a definitive population frame for individuals at risk of MASLD, which may affect the precision of representativeness. Further, it did not specifically address MetALD or ALD, which limits the ability to differentiate behavioral and clinical risk factors associated with varying degrees of alcohol consumption alongside metabolic dysfunction,^11^ and similarly restricts our capacity to isolate how alcohol intake may independently confound terminology awareness, health care utilization, and baseline levels of health concern.

## Supplementary Material

## References

[1] Lazarus JV Halpern B Schwarz PEH Boudou D Van Spall H Miller V . Global position statement supporting a World Health Assembly resolution on steatotic liver disease. Nat Rev Gastroenterol Hepatol. 2026;23:449–451. 10.1038/s41575-026-01201-z 41986753

[2] World Health Organization . Steatotic Liver Disease: A Missing Piece in the Global Noncommunicable Disease Response (EB158R1). 2026.

[3] Huang DQ Wong VWS Rinella ME Boursier J Lazarus JV Yki-Järvinen H . Metabolic dysfunction-associated steatotic liver disease in adults. Nat Rev Dis Primers. 2025;11:14. 10.1038/s41572-025-00599-1 40050362

[4] Alp J Sripongpun P Roldan GA Fletcher JA Davie T Udompap P . Low awareness of MASLD among U.S. adults: Trends from NHANES 2017–2023. Hepatol Commun. 2025;9:e0829. 10.1097/HC9.0000000000000829 PMC12520227 41085552

[5] Bril F Bolla P Huynh K, Endocrine SBJ of the, 2025 undefined . Prevalence of MASLD in people with diabetes has decreased in the US, but rates of liver fibrosis are still on the rise. *academic.oup.com*. 2025. doi:10.1210/JENDSO/BVAF110/8162705 PMC12210375 40599337

[6] Lazarus JV White TM Allen A Pannain S Alkhouri N Bansal MB . Awareness of metabolic dysfunction-associated steatotic liver disease (MASLD) in four major cities in the United States. Hepatol Commun. 2026;2:102200. 10.1097/HC9.0000000000000704 PMC12055070 40331869

[7] Pugliese N White TM Brennan PN Pannain S Hagström H Michel M . Diagnostic innovation and models of care to improve fibrosis detection and risk stratification in steatotic liver disease. Lancet Reg Health Eur. 2026;65:101722. 10.1016/j.lanepe.2026.101722 PMC13245525 42266906

[8] Lazarus JV White TM Manolas MI Allen AM Pannain S Alkhouri N . Awareness of metabolic dysfunction-associated steatotic liver disease (MASLD) among primary care providers in the four most populous U.S. cities. Ann Hepatol. 2026;31:102200. 10.1016/j.aohep.2026.102200 41825781

[9] Rinella ME Lazarus JV Ratziu V Francque SM Sanyal AJ Kanwal F . A multisociety Delphi consensus statement on new fatty liver disease nomenclature. J Hepatol. 2023;79:1542–1556. 10.1016/j.jhep.2023.06.003 37364790

[10] Lazarus JV Agirre-Garrido L White TM Arora AK Manolas MI Diaz LA . Best buys for metabolic dysfunction-associated steatotic liver disease and metabolic dysfunction-associated steatohepatitis: A global Delphi study. Lancet Gastroenterol Hepatol. 2025;11:232–242. 10.1016/S2468-1253(25)00300-0 41397405

[11] Parker R Arab JP Lazarus JV Bataller R Singal AK . Public health policies to prevent alcohol-related liver disease. Nat Rev Gastroenterol Hepatol. 2025;22:587–594. 10.1038/s41575-025-01084-6 40461606

