# Supplementary material for: Gaps in awareness of metabolic liver disease among high-risk populations in the United States
Source: Hepatol Commun. 2026 Oct 19;10(11):e1065. doi: 10.1097/HC9.0000000000001065 (PMC13682830; doi:10.1097/HC9.0000000000001065)
Supplement: Supplementary file 1 [file hc9-10-e1065-s001.docx]

**Supplemental Materials for White TM, et al.**

**“Gaps in Awareness of Metabolic Liver Disease Among High-Risk Populations in the United States”**

**Supplemental Methods**

*Sampling Frame Approach*

Given the absence of a definitive population frame for individuals who are overweight or have diabetes, YouGov employed its “Census click” sampling methodology. This approach aims to approximate representativeness by leveraging demographic benchmarking and advanced weighting techniques, even when the exact distribution of the target population across sociodemographic characteristics is not fully known. YouGov employed its standard matching algorithm to align survey respondents with the constructed sampling frame. This approach ensures that the distribution of key demographic variables in the sample approximates that of the target population.

*Weighting and Propensity Score Adjustment*

To adjust for potential selection bias, matched cases were weighted to the sampling frame using propensity score methods. A logistic regression model was estimated to predict inclusion in the sampling frame, combining both matched cases and frame observations. The propensity score model included the following covariates: age, gender, race/ethnicity, education level, and home ownership. Estimated propensity scores were grouped into deciles based on their distribution in the sampling frame. Post-stratification was conducted across these deciles to align the sample more closely with the frame. The weighted dataset of 2,581 respondents was restricted to the analytic subsample of 1,000 individuals who were overweight or had diabetes. Final survey weights were trimmed to reduce the influence of extreme values and recentered to a mean of 1.

**Supplemental Table 1. Participant Characteristics**

| **Characteristic** | **% of N=1000** |
| --- | --- |
| *Gender* |  |
| Male | 49% |
| Female | 51% |
| *Race* |  |
| White | 64% |
| Black | 12% |
| Hispanic | 12% |
| Asian | 3% |
| Native American | 2% |
| Two or more races | 4% |
| Other | 4% |
| *Education* |  |
| Post-graduate | 12% |
| 4-year college | 21% |
| 2-year college | 10% |
| Some college | 19% |
| High school graduate | 31% |
| No high school | 7% |
| *Home ownership* |  |
| Own | 67% |
| Rent | 32% |
| Other | 1% |
| *Health insurance* |  |
| Insurance through a current or former employer or union, or insurance through a spouse's/ other family member's employer | 34% |
| Medicare | 28% |
| Medicaid | 24% |
| Insurance purchased directly from an insurance company | 8% |
| Any other type of health insurance or health coverage plan | 5% |
| TRICARE | 3% |
| VA | 3% |
| Indian Health Service | 1% |
| Any other type | 5% |
| No health insurance | 12% |
| *Employment Status* |  |
| Full-time | 32% |
| Retired | 23% |
| Unemployed | 11% |
| Permanently disabled | 11% |
| Part-time | 10% |
| Homemaker | 6% |
| Student | 3% |
| Temporarily laid off | <1% |
| Other | 3% |
| *Marital Status* |  |
| Married | 43% |
| Never married | 31% |
| Divorced | 10% |
| Widowed | 6% |
| Domestic/civil partnership | 6% |
| Separated | 4% |
| *MASLD risk factor for inclusion* |  |
| Diagnosed with pre-diabetes or T2D | 42% |
| BMI 30+ | 80% |

**Supplemental Table 2. Health concerns by sub-samples**

| *Terms* | *Full sample (n=1,000)* | *BMI 30+ (n=804)* | *Living with type 2 diabetes or prediabetes (n=441)* | *Reported MASLD diagnosis (n=70)* | *Knowing Parent with MASLD (n=18)* | *Knowing Partner with MASLD (n=22)* | *Knowing Friend with MASLD (n=62)* | *Knowing Colleague with MASLD (n=9)* |
| --- | --- | --- | --- | --- | --- | --- | --- | --- |
| Obesity | 57% | 64% | 51% | 69% | 45% | 81% | 63% | 33% |
| High cholesterol | 39% | 38% | 47% | 42% | 33% | 32% | 49% | 23% |
| Hypertension | 48% | 48% | 53% | 54% | 40% | 48% | 49% | 13% |
| Diabetes | 48% | 41% | 81% | 59% | 38% | 57% | 67% | 43% |
| Chronic kidney disease | 16% | 15% | 19% | 27% | 11% | 11% | 25% | 13% |
| Liver disease | 13% | 13% | 14% | 39% | 10% | 14% | 19% | 23% |
| Cardiovascular disease | 29% | 27% | 32% | 39% | 27% | 26% | 43% | 28% |
| Cancer | 28% | 29% | 28% | 27% | 18% | 27% | 28% | 34% |
| None | 13% | 15% | 4% | 7% | 22% | 4% | 6% | 16% |

Note: Question asked “When thinking about your health, which, if any, of the following conditions concern you? (check all that apply)”
